# Giant Posttraumatic Angiolipoma of the Forearm: A Case Report and Review of the Literature

**DOI:** 10.1155/2021/4047777

**Published:** 2021-07-21

**Authors:** Athanasios Fotiadis, Petros Ioannidis, Ioannis Skandalos, Stergios Papastergiou, Aristeidis Vrettakos, Theodoros Tzigkalidis, Themistoklis Vampertzis

**Affiliations:** ^1^Orthopaedic Surgery and Traumatology-Unit for Sport Injuries, Agios Pavlos General Hospital of Thessaloniki, 55134 Thessaloniki, Greece; ^2^Surgical Department, Agios Pavlos General Hospital of Thessaloniki, 55134 Thessaloniki, Greece; ^3^Department of Pathology, Agios Pavlos General Hospital of Thessaloniki, 55134 Thessaloniki, Greece; ^4^Trauma and Orthopaedics, Bedford Hospital NHS Foundation Trust, MK42 9DJ Bedford, UK

## Abstract

Angiolipoma is a type of lipoma, a benign soft tissue tumor. It is distinguished by the excessive degree of vascular proliferation and the presence of mature adipocytes. It occurs commonly on the trunk and extremities. Angiolipomas larger than 4 cm are classified as “giant,” and due to their size, histological evaluation is necessary to exclude malignancy. We report a case of a male patient who suffered from a giant noninfiltrating intramuscular angiolipoma which formed after venipuncture in the antecubital fossa. Clinical examination showed a palpable painless soft mass. Computed tomography (CT) and magnetic resonance imaging (MRI) demonstrated a giant angiolipoma on the right forearm. Surgical removal of the mass was performed, and the biopsy was negative for malignancy. To the best of our knowledge, this is the first report in the literature of posttraumatic intramuscular angiolipoma. Physicians and orthopedic/general surgeons should be aware of the possibility of soft tissue masses in a posttrauma situation.

## 1. Introduction

Angiolipomas are benign soft tissue tumors of mesenchymal origin which are made of mature adipocytes with an excessive degree of vascular proliferation [1]. They can occur in every part of the body, but they are more common on the extremities, trunk, head, and neck. They are often asymptomatic and painless except when they cause a mass effect [2]. Appropriate imaging is useful to depict the nature and the margins of the lesion, while histological evaluation is necessary for the correct diagnosis [3]. The pathogenesis of angiolipomas is still unclear, but acute or recurrent trauma has been suggested as a possible etiologic factor [1, 4, 5].

The authors present a case of a giant angiolipoma of the forearm in a male patient who reported a venipuncture in the affected area, 12 months before our clinical evaluation. To our knowledge and after reviewing the current literature, there are no confirmed cases of posttraumatic intramuscular angiolipoma.

## 2. Case Presentation

A 62-year-old male farmer presented in the outpatient clinic with a large mass on the volar surface of his right forearm (dominant arm). The patient reported that the mass started appearing right after a venipuncture in the region of the antecubital fossa, about 12 months ago. Since then, the mass gradually increased in size with a more rapid growth over the past 6 months. The patient complained of slight discomfort during flexion of the elbow. No history of previous trauma in the affected area was reported. Past medical history included arterial hypertension. In addition, he was a social drinker and a nonsmoker.

**Figure 1:**
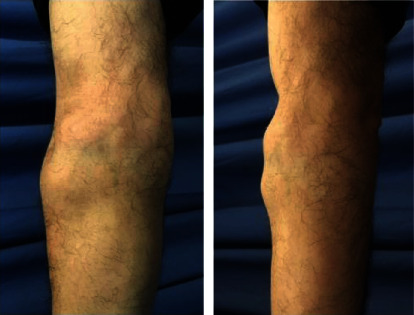
Preoperative view of the patient's forearm.

Physical examination revealed a large, palpable, and painless soft solid mass on the upper half of the right forearm (Figure 1). No overlying skin changes were found. Range of motion of the elbow, forearm, and wrist was normal. There were no sensory or motor defects, and peripheral circulation was normal. X-rays were negative for bone pathology. Blood tests were normal.

**Figure 2:**
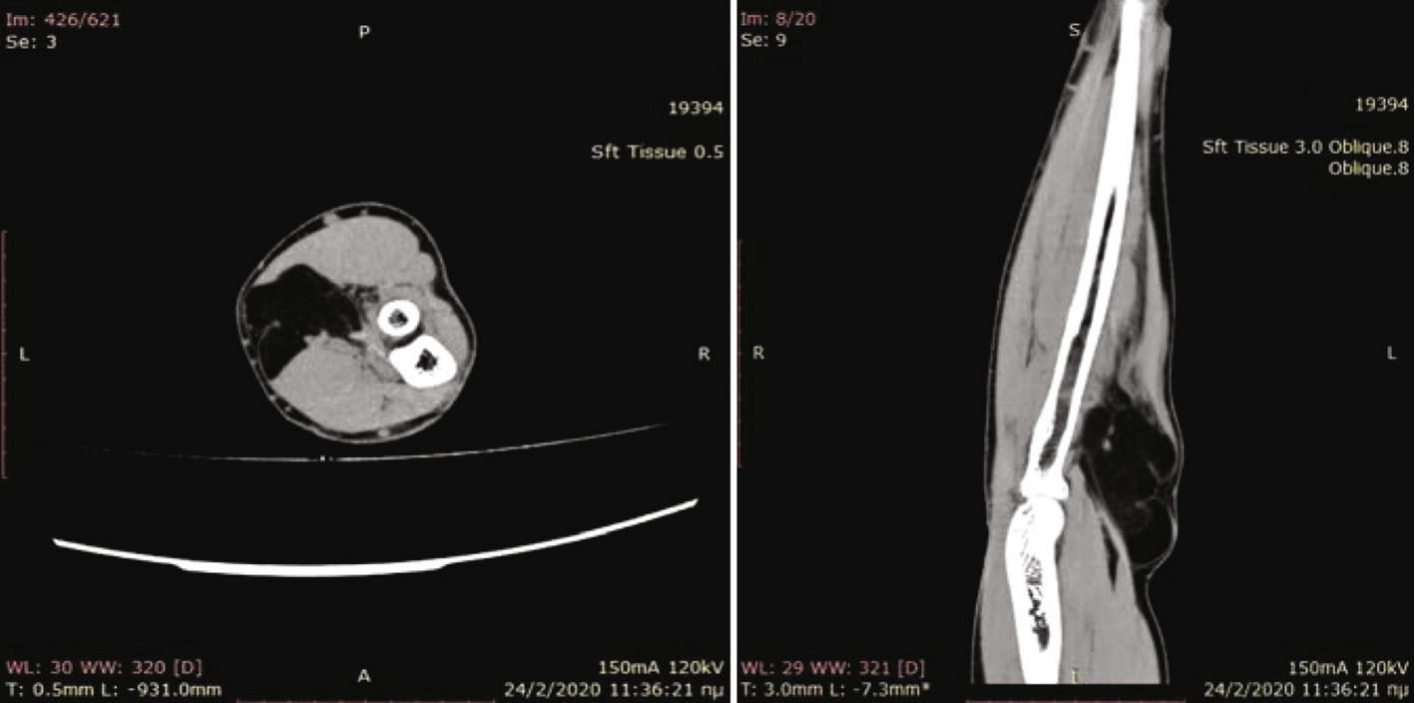
CT scans.

**Figure 3:**
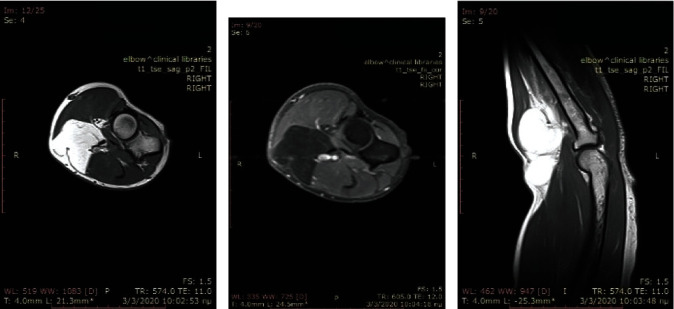
MRI images (performed with a 1.5-Tesla unit): (a) axial T1-weighted image; (b) axial T1 fat suppression; (c) sagittal T1-weighted image.

A CT and MRI with contrast demonstrated a well-encapsulated, solid multilobular lesion with lipoid content and the presence of septa (Figures 2 and 3). The enhancement of the septa demonstrated the rich vascularity of the lesion. There were no signs of infiltration of the surrounding tissues. The dimensions of the lesion were 4.8 × 4.7 × 7.03 cm. The findings suggested a lipoma, but the imaging could not exclude a sarcomatous transformation due to the presence of septa and of the dishomogeneity of the signal's density.

**Figure 4:**
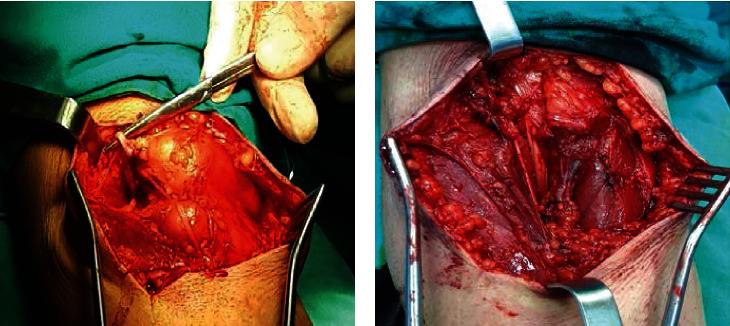
(a) Intraoperative view of the lesion and (b) residual cavity after the excision of the angiolipoma.

A lazy-S incision was performed, and the mass was removed surgically. The lesion occupied almost the entire upper half of the forearm including the antecubital fossa. It was located subcutaneously above the radial artery near the bifurcation of the brachial artery and between the brachioradialis and pronator teres muscles (Figure 4). The margins of the lesion were marked with metallic clips to determine the margins in case of malignancy and radiation therapy.

**Figure 5:**
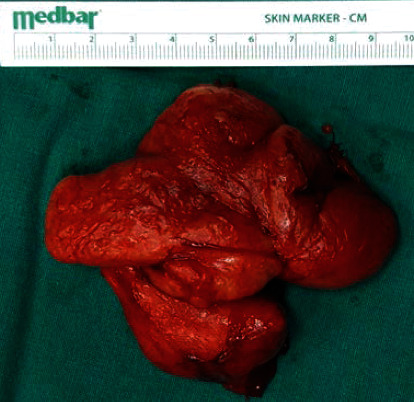
Macroscopic appearance of the surgical specimen.

**Figure 6:**
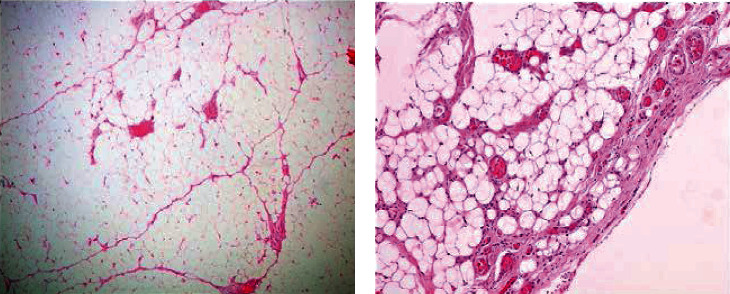
Histological examination of the lesion: (a) mature adipose and proliferated vascular tissue (hematoxylin and eosin; magnification ×40); (b) hyperplastic vessels with fibrous thrombi (hematoxylin and eosin; magnification ×100).

The removed mass's dimensions were 5 × 5 × 8 cm, and its weight was 68 grams (Figure 5). Histological examination showed mature adipose tissue with a vaguely lobular architecture, due to areas of excess fibrin deposition in the stroma (Figure 6(a)). Additionally, in a few subcapsular areas, there were evident small groups of tiny thin-walled hyperplastic vascular vessels, most of which exhibited the formation of fibrous thrombi in their lumens (Figure 6(b)). These morphological characteristics were compatible with the diagnosis of angiolipoma [6].

12 months postoperatively, the patient was asymptomatic.

## 3. Discussion

Although Bowen in 1912 first described a case of angiolipoma as a different entity from the generic diagnostic term “lipoma” [7], it was Howard and Helwig who established angiolipoma as an entity in 1960, describing it as a benign encapsulated and lobulated tumor that differs from lipoma by the presence of an excessive degree of vascular proliferation [1]. In 1966, J. J. Lin and F. Lin described 2 entities in angiolipomas, the infiltrating and noninfiltrating (encapsulated) types that should be treated differently because of their different biological behavior [2]. Their criteria to determine angiolipoma were as follows: (1) gross evidence of tumor formation with or without a capsule, (2) microscopic evidence of mature lipocytes as the major population (at least 50%) of the tumor, and (3) microscopic evidence of angiomatous proliferation inside the tumor.

At present, the WHO Classification of Soft Tissue Tumors classifies angiolipomas in the group of adipocytic tumors, the largest group of mesenchymal tumors (the same as lipomas, liposarcomas, etc.) [8]. It is described as a benign soft tissue tumor subdivided into infiltrative and noninfiltrative types. The rare infiltrative, nonencapsulated type typically involves deep soft tissues and is separated from the cutaneous (encapsulated) lesion because of its growth pattern and tendency to recur locally: this lesion is now best classified as intramuscular hemangioma [9].

Angiolipomas are more common in young adults (infiltrating angiolipomas are usually diagnosed in older patients), they are equally distributed between sexes and occur mostly in the extremities (2/3 of the cases in the forearm), trunk, spinal axis, head, and neck, and their size almost never exceeds 4 cm [5, 10]. Multiple lesions are seen in approximately 70-80% of cases, and 5% of these are familiar but with an unclear genetic pattern [5]. A recent study suggests an involvement of chromosome 13 in angiolipomas [11]. Clinically, the encapsulated/noninfiltrative angiolipoma presents as a subcutaneous nodule: the lesions are commonly multiple, typically firm, tender to palpation but often painful, and rarely associated with overlying skin changes [2, 5, 9, 10]. Pain and associated neuropathies are secondary to vascular engorgement and edema that can lead to compression of the adjacent neural tissue [10]. Because of the subcutaneous location, their—usually—small size, and their indolent clinical appearance, angiolipomas are often misdiagnosed as ordinary small lipomas and rarely imaged [12].

The diagnosis can be aided by ultrasonographic analysis (US), computed tomography (CT), or magnetic resonance imaging (MRI), but the microscopic examination is necessary for a conclusive diagnosis. US analysis is not very specific but can be useful for differentiating subcutaneous angiolipomas from ordinary lipomas by observing color Doppler flows [12]. CT contrast scans show a central low-density mass (lipomatous component) surrounded by areas of contrast enhancement (vascular component); studies without contrast show the homogenous low attenuation of a typical lipoma and may not define the margins and, therefore, the exact extent of the lesion [13]. Recent studies have shown that MRI with contrast defines well the margins of the lesion and demonstrates the presence of septa and the enhancement reveals the dense capillary proliferation. These results suggest that MRI is a useful noninvasive method in diagnosing and evaluating angiolipomas [3].

Microscopic examination of the surgical specimen is necessary for the conclusive diagnosis. The histopathologic characteristics were described by J. J. Lin and F. Lin as encapsulated (noninfiltrating angiolipomas) or nonencapsulated (infiltrating angiolipomas) lesions with, at least, 50% evidence of mature adipocytes and angiomatous proliferation in the tumor, in various ratios [2]. The presence of fibrinous microthrombi is a distinctive feature that differentiates angiolipomas from other lipomas. Angiolipomas have not been found to undergo malignant transformation [1, 5], but histological evaluation is necessary if measuring larger than 5 cm in a single dimension [14, 15].

The differential diagnosis of angiolipomas include lipomas and other lipoma variants (lipomatosis, myolipoma, chondroid lipoma, hibernoma, spindle-cell lipoma, atypical lipoma, pleomorphic lipoma, and lipoblastoma), hemangiomas, benign lesions affecting bone, joints, or tendons (intraosseous lipoma, parosteal lipoma, lipoma of joint or tendon sheath, and lipoma arborescens), and liposarcoma [5, 10].

The treatment of noninfiltrating angiolipomas is simple surgical excision, and they show no tendency to recur. Fine-needle aspiration is rarely diagnostic. Treatment of infiltrating angiolipomas consists of wide surgical excision, but there is a recurrence rate of 50%. When it is not associated with pain or has a small size (under 5 cm), it can be followed up without surgical treatment [3, 14].

The pathogenesis of this lesion remains unclear, and the origin of angiolipomas is still controversial. Increased familiar incidence has been reported (approximately 5%) but does not exceed that of ordinary lipomas [5]. Howard and Helwig and J. J. Lin and F. Lin suggest that angiolipomas originate as congenital lipomas that undergo vascular proliferation after further stimuli such as trauma [1, 2].

The relationship between trauma and formation of posttraumatic “tumors” has been investigated since 1935, and Ewing suggested that repeated or acute trauma could, rarely, cause a soft-tissue tumor or aggravate a preexisting one [4]. Since then, various authors have reported cases of posttraumatic lipomas, and still to date, their genesis remains unclear.

In their most recent study of 31 cases with posttraumatic lipomas and review of the literature, Aust et al. found an elevated PTT in their patients and reported a link between manifest or subclinical coagulation disorders and development of posttraumatic lipomas that are defined as adipose tissue tumors forming at the location of a precedent trauma, with a marked gender predominance towards women. They also reviewed precedent studies and possible theories on their formation [16]. The first “mechanical effect” of trauma theory [17, 18], suggests that acute or repeated trauma leads to a prolapse of adipose tissue through Scarpa's fascia forming a pseudolipoma (not a true lipoma because it is not encapsulated). The second theory based on studies with encapsulated lipomas without lesions in Scarpa's fascia [19–21] suggests that the induction of the differentiation of mesenchymal precursors (preadipocytes) to mature adipocytes and the effect of local/systemic inflammation and hormonal factors (such as growth hormones and sex steroids) can induce the formation of posttraumatic lipomas.

Posttraumatic angiolipomas are a very rare clinical diagnosis. With their case report and after review of the literature, Morgan et al. reported—in total—2 cases of cranial intraosseous angiolipomas, a very rare benign tumor of the bone, with a possible association with head trauma [22].

## 4. Conclusion

Angiolipomas are benign tumors of soft tissue composed of mature adipocytes with an excessive degree of vascular proliferation. They occur more commonly on the extremities and trunk/head of young adults, and their size rarely exceeds 4 cm. Among imaging tools, MRI with contrast is the most sensitive imaging tool to better define the lesion. Histological analysis is necessary to define the diagnosis, and surgical excision is the treatment of choice, especially in the presence of giant lesions. In the past years, more studies suggest that trauma can induce the formation of lipomas and various mechanisms were presented trying to explain the formation of posttraumatic lipomas. Physicians and surgeons should be aware of the possibility and recognize the formation of soft tissue masses in a posttrauma situation. To our knowledge and after reviewing the current literature, there are no confirmed cases of posttraumatic intramuscular angiolipomas.
